# Effects of Nandrolone in the Counteraction of Skeletal Muscle Atrophy in a Mouse Model of Muscle Disuse: Molecular Biology and Functional Evaluation

**DOI:** 10.1371/journal.pone.0129686

**Published:** 2015-06-11

**Authors:** Giulia Maria Camerino, Jean-François Desaphy, Michela De Bellis, Roberta Francesca Capogrosso, Anna Cozzoli, Maria Maddalena Dinardo, Roberta Caloiero, Kejla Musaraj, Adriano Fonzino, Elena Conte, Catherine Jagerschmidt, Florence Namour, Antonella Liantonio, Annamaria De Luca, Diana Conte Camerino, Sabata Pierno

**Affiliations:** 1 Section of Pharmacology, Dept. of Pharmacy & Drug Sciences, University of Bari “Aldo Moro”, Bari, Italy; 2 Galapagos-SASU, Romainville, France; Cinvestav-IPN, MEXICO

## Abstract

Muscle disuse produces severe atrophy and a slow-to-fast phenotype transition in the postural Soleus (Sol) muscle of rodents. Antioxidants, amino-acids and growth factors were ineffective to ameliorate muscle atrophy. Here we evaluate the effects of nandrolone (ND), an anabolic steroid, on mouse skeletal muscle atrophy induced by hindlimb unloading (HU). Mice were pre-treated for 2-weeks before HU and during the 2-weeks of HU. Muscle weight and total protein content were reduced in HU mice and a restoration of these parameters was found in ND-treated HU mice. The analysis of gene expression by real-time PCR demonstrates an increase of MuRF-1 during HU but minor involvement of other catabolic pathways. However, ND did not affect MuRF-1 expression. The evaluation of anabolic pathways showed no change in mTOR and eIF2-kinase mRNA expression, but the protein expression of the eukaryotic initiation factor eIF2 was reduced during HU and restored by ND. Moreover we found an involvement of regenerative pathways, since the increase of MyoD observed after HU suggests the promotion of myogenic stem cell differentiation in response to atrophy. At the same time, Notch-1 expression was down-regulated. Interestingly, the ND treatment prevented changes in MyoD and Notch-1 expression. On the contrary, there was no evidence for an effect of ND on the change of muscle phenotype induced by HU, since no effect of treatment was observed on the resting gCl, restCa and contractile properties in Sol muscle. Accordingly, PGC1α and myosin heavy chain expression, indexes of the phenotype transition, were not restored in ND-treated HU mice. We hypothesize that ND is unable to directly affect the phenotype transition when the specialized motor unit firing pattern of stimulation is lacking. Nevertheless, through stimulation of protein synthesis, ND preserves protein content and muscle weight, which may result advantageous to the affected skeletal muscle for functional recovery.

## Introduction

Skeletal muscle atrophy is a clinical condition that develops as a consequence of various diseases and pathophysiological conditions, including cancer (cachexia), chronic inflammatory syndromes, AIDS, renal failure, sepsis or diabetes, as well as of aging (sarcopenia), bed rest, limb immobilization or space flight (disuse). Although the triggers of skeletal muscle atrophy may differ among the various conditions, the underlying mechanisms likely overlap to some extent [1]. Actually, the loss of muscle mass may be caused by a decline of anabolic events or by the activation of catabolic pathways or impaired regeneration. The major pathways leading to muscle breakdown are the ubiquitin-proteasome system [2, 3], the Ca^2+^-dependent calpains [4, 5], and the lysosomal cathepsins [6]. Often the activation of one or more of these pathways can concur to the muscle damage and the understanding of the involved pathways is crucial for the development of successful drugs or nutritional interventions that can attenuate wasting and weakness and improve muscle structure and function.

During hindlimb unloading (HU) in rodents, an accepted model of muscle disuse [7, 8], the reduction of gravitational loading predisposes to severe muscle atrophy and functional decline in terms of weakness and increased fatigability, which lead to altered motor performance. The anti-gravity slow-twitch muscles are the most affected, since in response to reduced neuromuscular activity, they remodel their biochemical and contractile properties toward a faster phenotype [9]. Remodeling involves activation of specific intracellular signaling pathways and consequent genetic re-programming, which may also contribute to the progressive muscle degradation [10]. In principle, muscle atrophy is an adaptive response of the organism to maintain the metabolic and energy homeostasis in adverse conditions. Nevertheless, this response might turn into an unfavorable and irreversible event when muscle catabolism is activated. Disuse atrophy in rat skeletal muscle has been found to be associated with a decreased muscle fiber CSA and decreased muscle mass and reduced force producing capacity [11, 12] as well as with a rapid increase in mRNA encoding for the ubiquitin protein ligases, atrogin and muscle ring finger protein (MuRF1), during the first few days of disuse [13]. Also mRNA encoding for cathepsins was increased in different models of muscle wasting [1]. On the other hand, only small changes in the mRNA levels of Ca^2+^-dependent proteases were observed during disuse atrophy [11].

Together with muscle atrophy, we already observed structural and functional modification in terms of change of myosin heavy chain (MHC) isoform distribution in soleus (Sol) muscle of HU rat and mouse. Accordingly, the expression and function of proteins involved in the control of muscle excitability, excitation—contraction coupling, energy metabolism, and contractile properties are changed toward those of fast phenotype [14, 15]. Indeed, compared to control Sol muscle, a lower cytosolic calcium level is found in the HU Sol muscle [8, 16]. Interestingly, an increase of activity and expression of the ClC-1 muscle chloride channel, the channel responsible for the resting chloride conductance (gCl) is observed in HU animals toward the value typical of fast muscles. As already shown, this is important for muscle function, since the PKC-regulated gCl is pivotal for sarcolemma electrical stability and for the fine tuning of the contractile properties [15, 17, 18]. The modification of gCl in HU Sol muscle precedes the changes in MHC isoforms, suggesting the pivotal role of this parameter as a key determinant in promoting the phenotype transition [14].

At the aim to find effective countermeasures, we have previously tested the role of drugs or natural compounds expected to ameliorate muscle atrophy and functional damage induced by HU. The effects of taurine and trolox, were tested to modulate calcium homeostasis and to contrast oxidative stress, while chronic IGF-1 overexpression was tested with the aim to stimulate anabolism. Quite surprisingly, all the three pharmacological approaches were able to prevent the phenotype transition to some extent but showed very limited effect on Sol muscle atrophy. Treatment of HU mice with the potent antioxidant trolox fully prevented the lipoperoxidation and gCl increase, partially counteracted the MHC isoform transition, but had no effect on atrophy. The sulfonic amino-acid taurine, known to modulate both calcium homeostasis and gCl [19], prevented changes in gCl, restCa, and MHC expression, but lacked of effect on Sol muscle atrophy in HU rats. Finally, by using transgenic IGF-1-overexpressing mice we found that the growth factor ameliorates muscle function in terms of resting gCl as well as of the contractile parameters. Although it prevented the increased expression of MuRF1, the growth factor did not counteracted the loss of muscle weight and the decrease of fiber CSA in HU mice.

In previous studies, different anabolic steroids have been proposed to improve muscle wasting in various disease conditions. For instance, testosterone or anabolic steroids reduce muscle mass loss caused by microgravity [20], immobilization [21], and spinal cord injury [22, 23]. Nandrolone (ND) was also found to be beneficial in the recovery of the modification of contractile responses due to HU in saponin-treated fibers of rats [24]. However, side effects may limit their use in therapy. Although ND is considered as a potent anabolic agent with a relatively favorable safety profile, it may display significant binding to the progesterone receptor, with associated side effects such as water retention, acne, and gynecomastia [25]. Oxandrolone, another clinically used anabolic compound, has been shown to have a lower virilizing activity with respect to other anabolic steroids [26, 27]. Yet oxandrolone is taken per os, a route of administration quite difficult to apply to HU mice with respect to the parenteral administration of nandrolone.

Thus, in the present study, we evaluated the effects of ND treatment in HU mice on muscle weight and protein content, as markers of atrophy, and functional parameters, including resting gCl, resting intracellular calcium levels, and isometric contraction parameters that are modified during the phenotype transition. In an attempt to individuate the mechanisms by which ND can exert positive effects, we measured the expression of genes correlated to anabolic and/or catabolic signaling pathways. The results indicate that ND prevented atrophy, but not muscle function, likely through the activation of protein synthesis and possibly through the involvement of Notch-1 signaling.

## Materials and Methods

### Animal care and hindlimb unloading of mice

Experiments were performed in accordance with the Italian Guidelines for the use of laboratory animals (d.Lgs 2014 n26), which conforms with the European Union Directive for the protection of experimental animals (2011/63/EU), and received approval from the Italian Ministero della Salute (D.M. n.133/2000-B). Adult male C57BL/6J mice (5–8 month-old) were purchased from Charles River (Calco, Italy). Mice were subdivided in 4 groups of 15–20 animals. HU group: the mice were housed individually for 4 weeks, and were hindlimb unloaded for the last 2 weeks. HU-V group: the mice were housed individually for 4 weeks, received the vehicle (corn oil in 10% ethanol) s.c. daily for 4 weeks, and were hindlimb unloaded for the last 2-weeks. HU-ND group: the mice were housed individually for 4 weeks, received 5 mg/kg Nandrolone Decanoate (ND) s.c., once a day, 6 days a week, for 4 weeks, and were hindlimb unloaded for the last 2-weeks. CTRL group: the mice were housed individually in control conditions for 4 weeks. Because of the unfeasibility to perform all the designed measurements on one muscle, the animals were randomly assigned to subgroups used to study the electrophysiological, functional, biochemical or molecular parameters in EDL and Sol muscles (resting ionic conductance and excitability, resting calcium level, contractile properties, real-time PCR, western blot, protein content). Muscle unloading was induced as previously described [12]. The HU animals were examined daily over the entire HU period for behavior, cleanliness, aspect of hairs and eyes, food and water consumption. Daily food intake was measured as the mean value of the quantity of commercial food (in grams) eaten each day over 14 days by the mice belonging to CTRL and HU groups. The mice were given a standard amount of food (10 g) each morning, and the remaining food was weighted on the morning of the following day. We obtained the daily intake by subtraction, and a mean daily intake over 14 days was calculated for each animal. The final result reports the mean ± SEM from n animals in each experimental group. At the end of the treatment period, mice were weighted and deeply anesthetized with intraperitoneal injection of urethane (1.2 g/kg body weight) to allow removing of hind limb skeletal muscles, which are the soleus (Sol), Extensor Digitorum Longus (EDL), and gastrocnemius (Gas). These muscles were used immediately for electrophysiological and functional studies or frozen in liquid nitrogen and stored at -80°C for further biochemical and gene expression analysis. After surgery, animals were euthanized by an overdose of urethane. All efforts were made to minimize animal suffering.

### Total protein content measurement

Total protein content (TPC) of Sol and EDL muscles was quantified using a Bio-Rad protein assay (Bio-Rad, Hercules, CA, USA). Total proteins were extracted by treating 100–200 mg of frozen mice tissues with 400 μl of 50 mM Tris—HCl, pH 6.8, 100 mM DTT, 2% SDS, and 10% glycerol. Muscle homogenates were centrifugated at 12000 rpm and the supernatant including the myofibrillar fraction was extracted [12, 28–30]. Proteins were quantified with the Bradford method (Bio-Rad) according to the supplier’s instructions.

### Electrophysiological studies: ion channel resting conductances measured by the 2-intracellular microelectrodes technique

Sol and EDL muscles were fixed by tendons to a glass rod immersed in normal physiological (NP) or chloride-free (CF) solution maintained at 30°C [14]. The cable parameters of myofiber sarcolemma were determined from the electrotonic potentials elicited by square wave hyperpolarizing current pulse of 100-ms duration, using two intracellular microelectrodes in current-clamp mode, as previously described [31]. The membrane conductance is calculated from the values of input resistance, space constants and time constant and assuming a myoplasmic resistivity of 140 Ωcm. The mean chloride conductance (gCl) is calculated as the mean total membrane conductance (gm) measured in NP solution minus the mean potassium conductance gK measured in chloride-free solution.

### Fluorescence measurements of resting cytosolic Calcium concentration (restCa)

The resting cytosolic Ca^2+^ concentration (restCa) was determined in freshly, mechanically dissected muscle fibers using a QuantiCell 900 fluorescence imaging system (Visitech International, Sunderland, UK), as previously described [32]. Briefly, small bundles of 5–10 fibers were dissected tendon to tendon from Sol, Gas, and EDL muscles. The bundles were incubated for 60–90 min at 30°C in NP solution supplemented with 5 μM of acetoxymethyl ester of Fura-2 mixed to 10% (v/v) pluronic F-127 (Molecular Probes, Leiden, The Netherlands). After incubation, the cells were washed with NP solution and mounted in a modified RC-27NE recording chamber (Warner Instruments, Hamden, CT). Tendons of the bundles were attached by hair loops, one extremity to a fixated tube, the other to a mobile one. The chamber was accommodated on the stage of an inverted Eclipse TE300 microscope equipped with a x40 pan-fluorobjective (Nikon, Tokyo, Japan). The recording chamber was continuously perfused with NP solution at a constant rate of ~4 ml/min. Fiber integrity was controlled by assessing contractile activity under×400 magnification in response to a depolarizing solution containing 100 mM K+. Then the mean sarcomere length was adjusted to ~2.5 μm. The mean resting background-corrected ratio (340 nm/380 nm) values were determined for each fiber of the bundle by manually demarcating fiber boundaries using QC2000 software. This fluorescence ratio was converted off-line to restCa by using the calibration parameters determined in each muscle, using the equation:

$$restCa\;=\;\frac{\left(R-R_{min}\right)}{\left(R_{max}-R\right)}K_{D}\beta$$

where R is the 340 to 380 nm fluorescence ratio; K_D_ is the affinity constant of Fura-2 for Ca^2+^ given by the manufacturer, i.e. 145 nM (Molecular Probes), and β, R_min_, and R_max_ are calibration parameters determined in ionomycin-permeabilized fibers bath and in NP solution for the calculation of R_max_ or in Ca^2+^-free solution for the calculation of R_min_ [33]. The β value was calculated as the ratio of fluorescence intensities emitted by the fibers excited at 380 nm in Ca^2+^-free and NP solutions. The calibration parameters were measured for every muscle types in each experimental condition, because they may be different between muscle types and may change critically as a function of muscle state [32].

### Isometric contraction measurements

The Sol and EDL muscles were removed from the anesthetized mice and placed in NP solution for 30 minutes to equilibrate, as previously described [34]. The muscles were placed in a recording chamber at 27 ± 1°C. One tendon was fixed to a force transducer (FORT25, WPI Inc, FL, USA) connected to proper interface and data acquisition units (MP100; Biopac Systems, CA, USA), while the opposite tendon was fixed to a chamber hook. Data were collected and analysed using AcqKnowledge software v. 3.8 (Biopac System). An electrical stimulation field was obtained with two axial platinum wires flanking the muscle and connected to a stimulator (LE12406, 2Biological instruments, VA, Italy). The muscles were stretched to the optimum length (Lo measured with calipers), which is the muscle length allowing the maximal isometric contraction in response to a square-wave pulse of 40 V and 0.2 ms. Then specific voltage stimulus were applied to elicit isometric contraction. The maximal twitch tension (Ptw, mN), time to peak (TTP, ms), and half-maximal relaxation time (HRT, ms) of the single twitch were calculated as the mean values from 5 single twitches recorded every 30 s. The maximal tetanic isometric contraction (Po, mN) was calculated from the force-frequency relationship constructed with trains of 0.2 ms-40 V pulses applied from 10 to 140 Hz; train duration was 1200 ms for Sol muscle and 350 ms for EDL muscle. The specific twitch tension (sPtw, kN/m^2^) and specific tetanic tension (sPo, kN/m^2^) were obtained by normalizing maximal force value with respect to the cross-sectional area calculated from the muscle mass and length [35, 36].

### Isolation of total RNA, reverse transcription and Real-time PCR

Sol and EDL muscles were snap frozen in liquid nitrogen soon after removal and stored at -80°C until use. For each muscle sample, total RNA was isolated by RNeasy Fibrous Tissue Mini Kit (Quiagen C.N. 74704) and quantified by using a spectrophotometer (ND-1000 NanoDrop, Thermo Scientific). To perform reverse transcription, for each sample, 400 ng of total RNA was added to 1 μl dNTP mix 10 mM each, (Roche N.C. 11277049001), 1 μl Random Hexamers 50 μM (life-technologies C.N. n808-0127) and incubated at 65°C for 5 min. Afterward, 4 μl 5X First Standard Buffer (life-technologies C.N. Y02321), 2 μl 0,1 M DTT (life-technologies C.N. Y00147) and 1μl Recombinant RNasin Ribonuclease Inhibitor 40 U/μl (Promega, C.N. N2511) were added and incubated at 42°C for 2 min. To each solution 1 μl Super Script II Reverse Trascriptase 200 U/μl (life-technologies C.N. 18064–014) was added and incubated at 25°C for 10 min, at 42°C for 50 min and at 70°C for 15 min. Real-time PCR was performed in triplicate using the Applied Biosystems Real-time PCR 7500 Fast system, MicroAmp Fast Optical 96-Well Reaction Plate 0.1 mL (life-technologies C.N. 4346906) and MicroAmp Optical Adhesive Film (life-technologies C.N. 4311971). Each reaction was carried in triplicate on a single plex reaction. The setup of reactions consisted 8 ng of cDNA, 0,5 μl of TaqMan Gene Expression Assays, (life-technologies), 5 μl of TaqMan Universal PCR master mix No AmpErase UNG (2x) (life-technologies C.N. 4324018) and Nuclease-Free Water not DEPC-Treated (life-technologies C.N. AM9930) for a final volume of 10 μl. Under the owing RT-TaqMan-PCR conditions: step 1: 95°C for 20 s; step 2: 95°C for 3 s; and step 3: 60°C for 30 s; steps 2 and 3 were repeated 40 times. The results were compared with relative standard curve obtained by 5 points of 1:4 serial dilutions. TaqMan Hydrolysis primer and probe gene expression assays were ordered by life-technologies with the following assay IDs: mammalian target of rapamycin, serine/threonine kinase (encoded by *mTOR* gene) assay ID: Mm00444968_m1; eukaryotic translation initiation factor 2 alpha kinase 3 (encoded by *Eif2ak3* gene) assay ID: Mm00438700_m1; peroxisome proliferative activated receptor, gamma, coactivator 1 alpha, PGC1a (encoded by *Ppargc1a* gene) assay ID: Mm01208835_m1; microtubule-associated protein 1 light chain 3 beta, LC3-β (encoded by *Map1lc3a* gene) assay ID: Mm00458725_g1; cathepsin L (encoded by *Ctsl* gene) assay ID: Mm00515597_m1; F-box protein 32, also called Atrogin1 (encoded by *Fbxo32* gene) assay ID: Mm00499523_m1; tripartite motif-containing 63 also called Murf-1 (encoded by *Trim63* gene) assay ID: Mm01185221_m1; myosin heavy chain 1 (encoded by *Myh7* gene) assay ID: Mm00600555_m1; myosin heavy chain 2a (encoded by *Myh2* gene) assay ID: Mm00454982_m1; myosin heavy chain 2b (encoded by *Myh4* gene) assay ID: Mm01332541_m1; myosin heavy chain 2x (encoded by *Myh1* gene) assay ID: Mm01332489_m1; notch1 (encoded by *Notch1* gene) assay ID: Mm00435249_m1; myogenic differentiation 1 (encoded by *MyoD* gene) assay ID: Mm00440387_m1; myogenic factor 5 (encoded by *Myf5* gene) assay ID: Mm00435125_m1; myogenin (encoded by *Myog* gene) assay ID: Mm00446194_m1; paired box 7 (encoded by *Pax-7* gene) assay ID: Mm01354484_m1; hypoxanthine guanine phosphoribosyl transferase (encoded by *Hprt1* gene) assay ID: Mm00446968_m1; actin, beta (encoded by *Actb* gene) assay ID: Mm00607939_s1; beta-2 microglobulin (encoded by *B2m* gene) assay ID: Mm00437762_m1. The mRNA expression of the genes was normalized to the best housekeeping genes hypoxanthine *Hprt1* selected from *Actb* and *B2m* and *Hprt1* by GenNorm software. For genes that are poorly expressed, such as *Myod*, *Myf5*, *Myog*, *Pax-7*, *Hprt1*, a pre-amplification by TaqMan PreAmp Master Mix (Life Technologies C.N. 4391128) was made before the real-time experiments. The set-up of pre-amplification consisted by 250 ng of reverse-transcribed (in 12.5 ml volume), 25 ml of TaqMan PreAmp Master Mix (2x) and 12.5 ml of pool assay 0.2 x (containing *MyoD*, *Myf5*, *Myog*, *Pax-7*, *Hprt1*). The solution was incubated at 50°C for 2 min, 95°C for 10 min and for 40 cycles of 95°C for 15 s and 60°C for 1 min. The methods of gene expression analysis are the same as those previously used [37]. The RT-PCR experiments were performed in agreement with the MIQE guidelines for qPCR, as published [38].

### Western blot analysis

Soleus muscles of each group were homogenized in 200 μl of lysis buffer (Tissue Protein Extraction Reagent, Thermo Scientific, cod. 78510), enriched with Phosphatase Inhibitor Cocktail (Sigma-Aldrich, cod. P5726) diluted 1:100 and Protease Inhibitor Cocktail (Sigma-Aldrich, cod. P8340) diluted 1:100. After incubation on ice for 30 minutes, the homogenate was collected by centrifugation at 12000 *rpm* for 10 minutes at 4°C. Protein concentration of homogenates was determined via the Bradford method (Bio-Rad Protein Assay, cod. 500–0006). Homogenated samples were boiled in a Laemmli 2x sample Buffer (Bio-Rad, cod. 161–0737) for 5 minutes. Thirty microgram of total protein of each sample was separated by SDS-PAGE (12% gels) and transferred to Immuno-Blot PVDF membranes (Bio-Rad, cod. 162–0177). Single SDS-PAGE gels were run for each sampled protein. Membranes were blocked with 5% Blotting-Grade Blocker (Bio-Rad, cod. 170–6404) in Tris Buffered Saline (TBS) containing 0,5% Tween-20 (TBST) for 2 hour and then incubated with primary antibody overnight at 4°C under continuous agitation. After two washing with TBST and one with TBS and water, the membranes were incubated with goat anti-rabbit second antibody (diluted 1:5000, Bio-Rad, cod. 170–5046) conjugating with horseradish peroxidise (HRP) for 1 hour at room temperature under continuous agitation. Protein bands were detected by ChemiDoc XRS (BioRad) following reaction with a solution 1:1 of Clarity Western ECL Substrate Luminol/enhancer solution (Bio-Rad, cod. 102030394) and Clarity Western ECL Substrate Peroxidase solution (Bio-Rad, cod. 102030396). The densitometric analysis was performed on the blot using Image Lab Analysis Software. The software allows the chemiluminescence detection of each experimental protein band to obtain the absolute signal intensity. The density volume was automatically adjusted by subtracting the local background. For each sample, the relative intensity was calculated by normalizing the experimental absolute intensity of target protein (eIF2α and phospho—eIF2α) with the intensity of β-actin protein band as reference standard. Primary antibodies against eIF2α (dilution 1:1000, cod. 9722) and phospho—eIF2α (Ser 51, dilution 1:1000, cod. 9721) were purchased from Cell Signaling Technology, while primary antibody against actin (dilution 1:200, cod. A2066) was purchased by Sigma-Aldrich.

### Drug and solutions

Corn oil and Nandrolone decaonoate were obtained from Galapagos-SASU. The dose of ND was formulated so to inject 0.1 ml/10 g body weight. The vehicle used to dissolve ND was corn oil in ethanol (10%). The normal physiological (NP) solution contained (in mM): NaCl 148, KCl 4.5, CaCl_2_ 2.5, MgCl_2_ 1.0, NaHCO_3_ 12.0, NaH_2_PO_4_ 0.44, glucose 5.5. The solution was gassed with 95% O_2_ and 5% CO_2_ to maintain the pH in between 7.2 and 7.4. The chloride-free (CF) solution was prepared by equimolar substitution of methylsulfate salts for NaCl and KCl and nitrate salts for CaCl2 and MgCl2. The calcium free-solution has the same composition as that of the normal physiological solution except that CaCl2 was omitted and 10 mM ethylene glycol bis (β-aminoethyl ether)- N,N,N’,N’-tetracetic acid (EGTA) was added. All chemicals were purchased from Sigma-Aldrich (Milano, Italy).

### Statistical analysis

For multiple comparison, statistical analysis was performed using one-way analysis of variance (ANOVA) followed by ad-hoc Bonferroni t-test or Fisher t-test. P values <0.05 were considered statistically significant.

## Results

### Effects of HU and Nandrolone on Muscle weight and Muscle-to-body weight ratio

**Fig 1:**
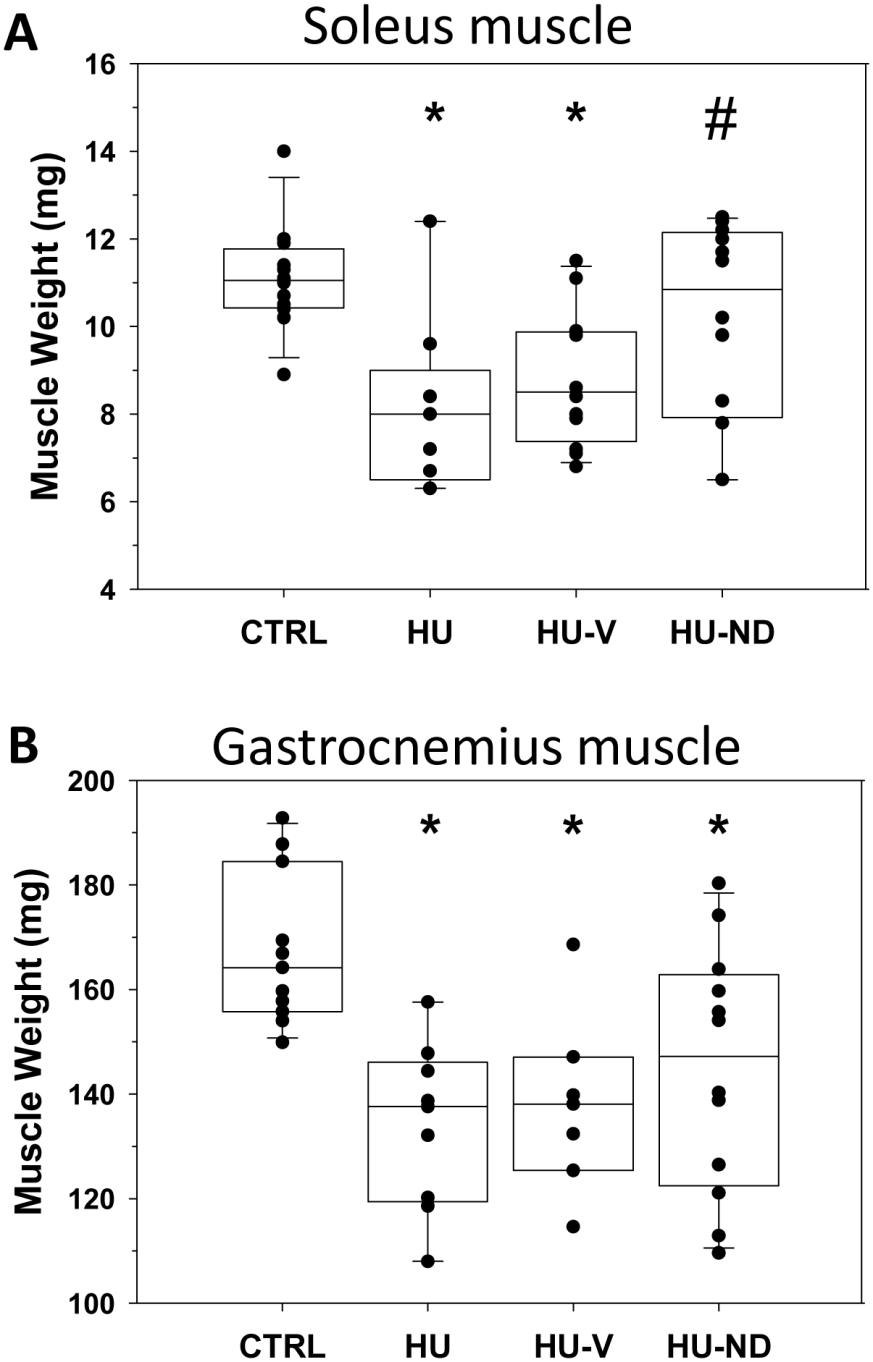
Effects of Nandrolone (ND) treatment on muscle weight of HU mice. (A) Box and whisker chart created on the basis of single Soleus muscle weight (circles) of 12 control mice (CTRL), 9 hindlimb unloaded (HU) mice, 12 vehicle-treated hindlimb unloaded (HU-V) mice; 12 nandrolone-treated hindlimb unloaded (HU-ND) mice. (B) Box and whisker chart created on the basis of single Gastrocnemius muscle weight (circles) of 11 control mice (CTRL), 9 hindlimb unloaded (HU) mice, 7 vehicle-treated hindlimb unloaded (HU-V) mice; 12 nandrolone-treated hindlimb unloaded (HU-ND) mice). Statistical analysis was performed for each muscle type using ANOVA followed by Bonferroni’s t-test (F = 5.5, dF = 3/41, P<0.003 for Sol muscle) (F = 6.6, dF = 3/35, P<0.002 for Gas muscle). Significantly different (at least P<0.05) versus (*) CTRL and versus (#) HU.

The HU animals did not show any sign of stress during the experimental period. Daily food consumption over 14 days did not differ significantly between control (n = 15) and HU (n = 11) mice (4.52 ± 0.14 g/day vs. 4.38 ± 0.15 g/day, respectively). The mouse body weight was significantly decreased after 14 days of HU with respect to controls and was preserved in HU-ND-treated mice (S2 Table). We examined the muscle weight in the different muscle phenotypes (Sol, Gas, EDL), which are characterized by a different amount of fast and slow fibers. As previously found in C57BL mice [12,39], the Sol muscle is composed by 39% of type I fibers, 50% type IIa and 11% IIx, whereas EDL and Gas muscles are quite exclusively composed of type IIb fibers, although the Gas muscle showed a presence of a little amount of type I and type IIa fibers not found in the EDL muscle. Fourteen-days HU induced a decrease in muscle weight (MW) in Sol and Gas muscles, indeed MW was significantly reduced by 27% and 20%, respectively, compared to controls. In average, Sol MW was significantly greater in HU-ND mice compared to HU mice (Fig 1). Actually, the HU-induced Sol MW drop was largely prevented in 58% of the mice (7 out of 12 mice had MW > 10 mg). The vehicle had no effect, since the muscle weight of HU-V mice was similar to that of HU mice and significantly lower than that of control (Fig 1). The averaged Gas MW of HU-ND mice was slightly recovered, although the value was still significantly lower than CTRL (Fig 1). Yet, similarly to Sol muscle, the distribution of MW values suggests a prevention of Gas atrophy in 50% of HU-ND mice. In contrast, the weight of EDL muscle was not modified in the four experimental conditions (S1 Fig). Similar modifications, although not significant, were found in Sol muscle weight-to-body weight ratio, being the CTRL value 0.37 ± 0.01 mg/g (n = 12), the HU value 0.31 ± 0.02 mg/g (n = 9), the HU-V value 0.29 ± 0.02 mg/g (n = 12) and the HU-ND value 0.34 ± 0.02 mg/g (n = 12).

### Effects of HU and Nandrolone on total protein content of mouse skeletal muscle

**Fig 2:**
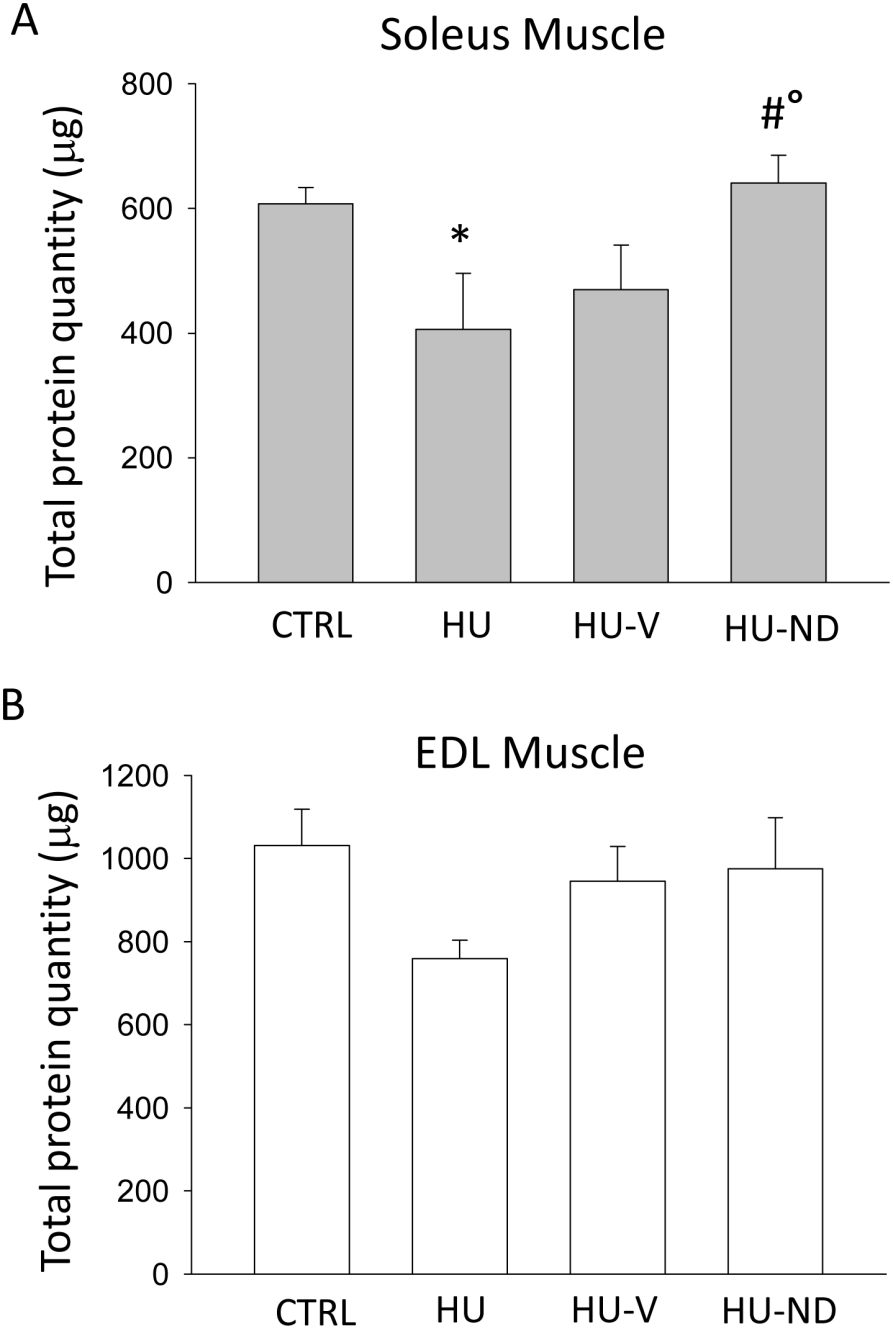
Effects of Nandrolone (ND) treatment on muscle total protein content of HU mice. Each bar represents the mean ± S.E.M. calculated from **(A)** 6 control (CTRL) Soleus (Sol) muscles; 6 hindlimb unloaded (HU) Sol muscles; 5 vehicle-treated hindlimb unloaded (HU-V) Sol muscles; 6 nandrolone-treated hindlimb unloaded (HU-ND) Sol muscles and **(B)** 6 control (CTRL) Extensor Digitorum Longus (EDL) muscle samples; 6 hindlimb unloaded (HU) EDL muscles; 6 vehicle-treated hindlimb unloaded (HU-V) EDL muscles; 6 nandrolone-treated hindlimb unloaded (HU-ND) EDL muscles. Statistical analysis was performed for each muscle type using ANOVA followed by Bonferroni’s t-test (F = 3.25, dF = 3/19, P<0.05 for Sol muscle) (F = 1.78, dF = 3/20, N.S. for EDL muscle). Significantly different (at least P<0.05) versus (*) CTRL, versus (#) HU and versus (°) HU-V.

In addition to muscle weight, which may depend on variation of protein content or water fluxes or fibrosis, we measured the total protein content in Sol and EDL muscles as an outstanding measure of atrophy. As observed in the Fig 2, HU induced a significant drop of protein content in Sol muscle (-33.1 ± 14.5%) with respect to control, which was however preserved by ND treatment (+37 ± 13.5% with respect to HU). As expected, the treatment with the vehicle did not ameliorate protein muscle level (-23 ± 11.2% with respect to CTRL). Similar trend was observed in EDL muscle although no statistical difference was found as assessed by ANOVA test (Fig 2).

### Effects of HU and Nandrolone on cable parameters and resting chloride conductance in mouse skeletal muscle

**Fig 3:**
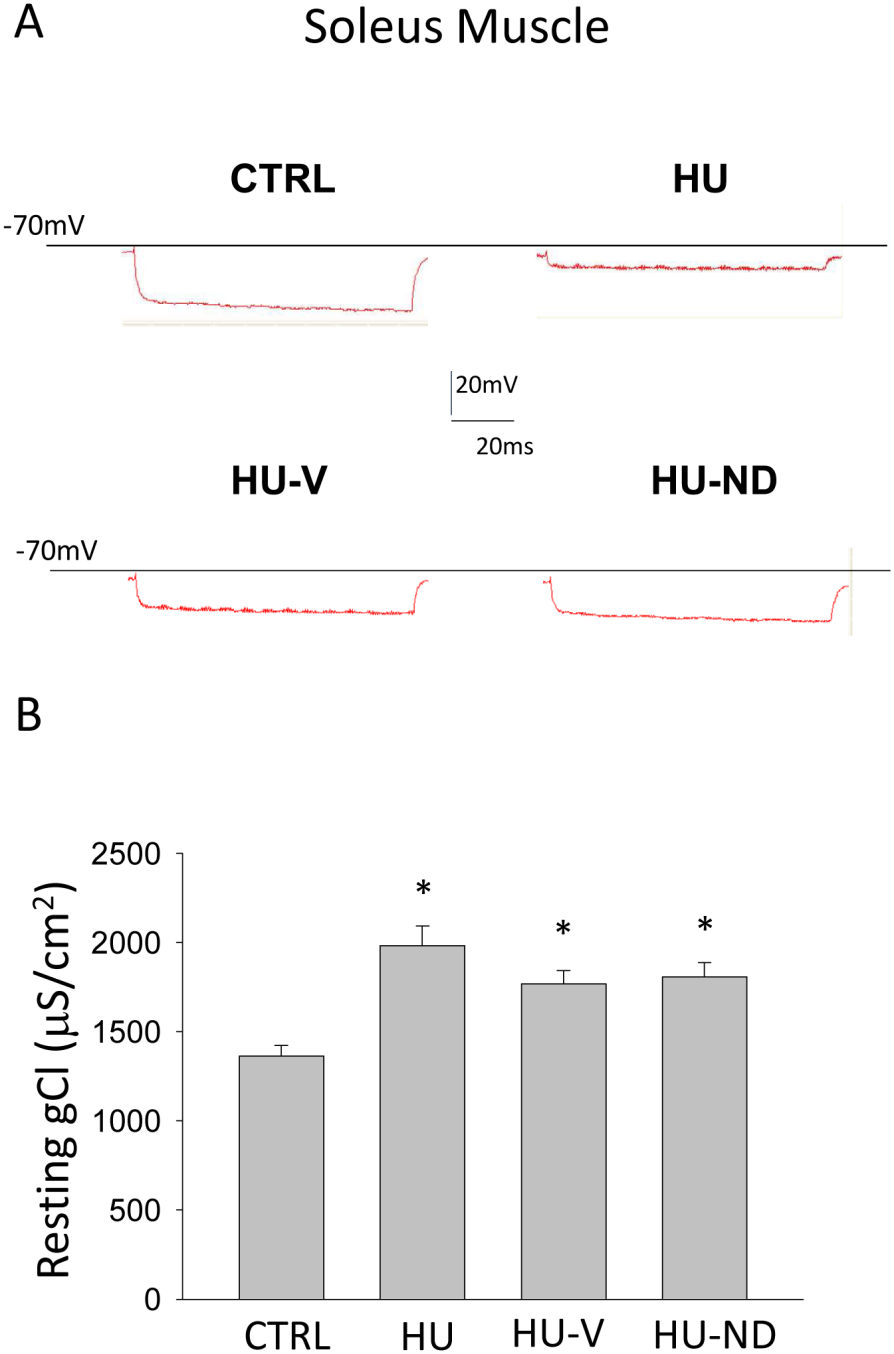
Effects of Nandrolone (ND) treatment on the resting chloride conductance (gCl) in Soleus muscle of HU mice. (**A**) Representative traces of the electrotonic potentials recorded in Sol muscle fibers by standard two microelectrodes technique at 0.05 mm distance between electrodes, in response to hyperpolarizing square-wave current pulse. The electrotonic potential recorded in normal physiological solution allows to measure membrane resistance Rm and its reciprocal, the total membrane conductance (gm). The electrotonic potential recorded in chloride-free solution allows to measure the potassium conductance (gK). The chloride conductance (gCl) is the mean gm minus the mean gK. (**B**) Measure of resting chloride conductance (gCl). Each bar represents the mean ± S.E.M. measured in Sol muscles from 3 CTRL animals/ 30 fibers; 3/20 HU; 6/44 HU-V; 5/44 HU-ND. Statistical analysis was performed for each muscle type using ANOVA followed by Bonferroni’s t-test (F = 8.05, dF = 3/134, P<0.001 for Sol muscle). Significantly different (at least P<0.05) versus (*) CTRL.

We previously demonstrated the involvement of resting chloride conductance (gCl), sustained by the ClC-1 channel, in HU-induced muscle disuse [8,12,14,15]. This channel strongly contributes to the modification of chloride ion permeability through the sarcolemma during HU. Accordingly, other channels were found to be involved in the modification of sarcolemma permeability and functional changes observed during denervation-induced atrophy in fast muscles [40]. Resting gCl is lower in the slow-twitch Sol muscle compared to the fast-twitch EDL muscle, and was found to be increased after 14 days HU in Sol muscle toward the fast value as a result of slow-to-fast transition [14]. Accordingly, here we found a significant increase of gCl by 45.5 ± 7.4% in Sol muscle of HU mice (Fig 3). No significant effect of vehicle or ND treatment was observed on gCl of HU mice (+30 ± 5.5% and +33 ± 6.0%, respectively, as compared to CTRL) (Fig 3). Indeed, among the six vehicle-treated mice tested for gCl, 5 animals showed a gCl value in Sol muscle similar to that of HU animals (gCl>1600 μS/cm^2^), while 1 mouse had a gCl more similar to that of controls (gCl<1600 μS/cm^2^). Within the ND-treated group, 4 mice showed a high Sol muscle gCl (HU-like) and 1 showed a low gCl (control-like). The value of gCl in each single animal showed no correlation with the level of muscle atrophy (data not shown). During previous studies [8, 14], muscles with control-like gCl were observed with a similar occurrence in HU rats. Regarding the EDL muscle, no significant difference was found between vehicle-treated and ND-treated mice, which showed values very similar to both control and HU animals (S2 Fig).

### Effects of HU and Nandrolone on the resting cytosolic calcium concentration (restCa) in mouse skeletal muscle

**Fig 4:**
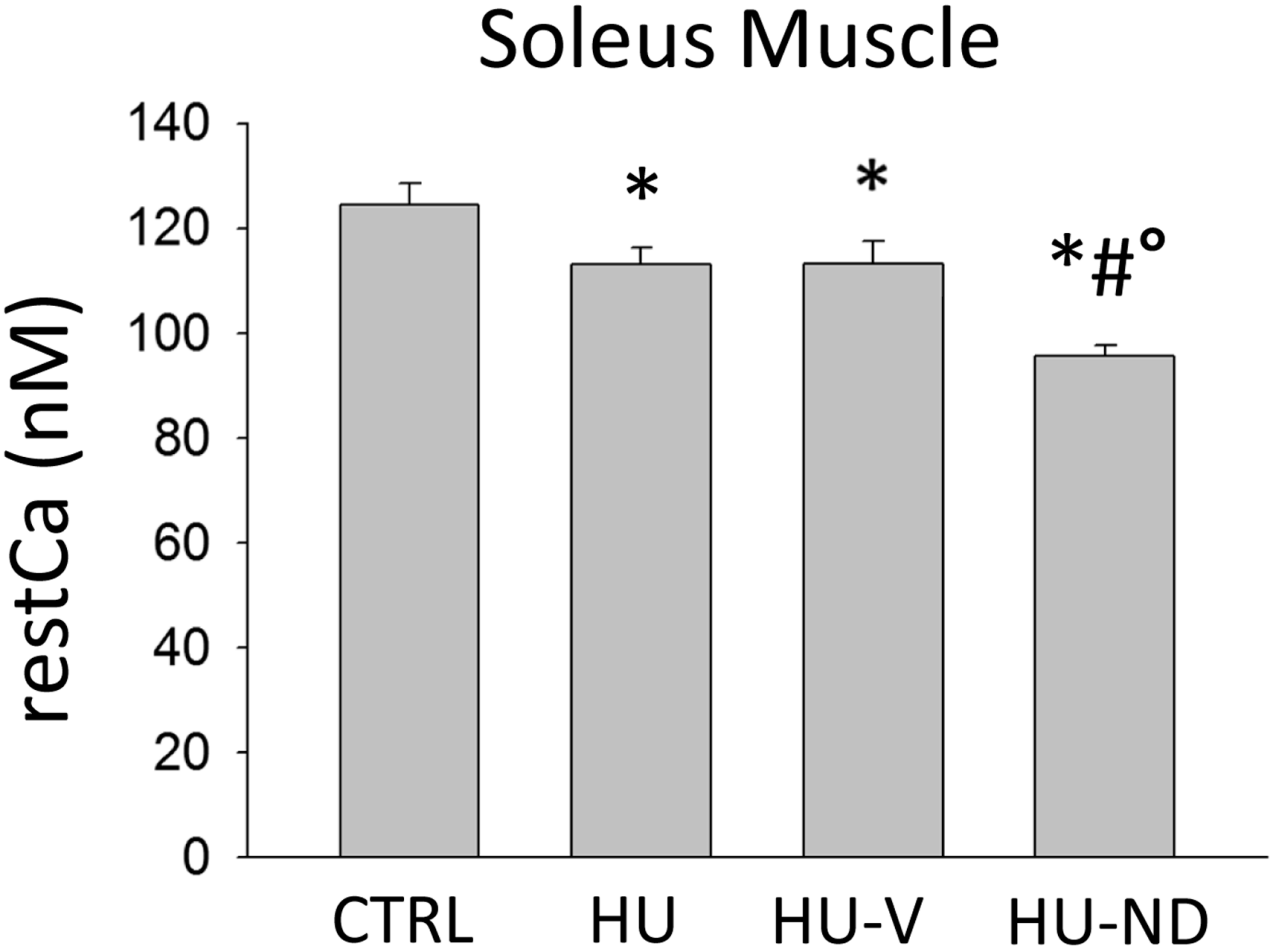
Effects of Nandrolone (ND) treatment on resting calcium (restCa) level in Soleus muscle of HU mice. Each bar represents the mean ± S.E.M. measured in the Sol muscles from n fibers/ of N animals in the different experimental conditions: CTRL 47/4; HU 58/4; HU-V 50/6; HU-ND 78/6. Statistical analysis was performed for each muscle type using ANOVA followed by Bonferroni’s t-test (F = 13.7, dF = 3/229, P<0.001 for Sol muscle). Bonferroni’s t-test showed significant difference (at least P<0.05) versus (*) CTRL, (#) HU, (°) HU-V.

The resting cytosolic Ca^2+^ concentration (restCa) is a fiber type-dependent parameter [16], being greater in slow-twitch compared to fast-twitch muscles. In rats and mice, the restCa decreases in Sol muscle after HU in accord with the slow-to-fast transition of the muscle [12, 16]. Accordingly, in this set of experiments, restCa was slightly but significantly reduced in Sol muscles of HU mice (4–6 animals for each experimental condition were used) (Fig 4). No significant effect of vehicle treatment was observed, whereas ND treatment further significantly decreased the restCa in Sol muscle of HU-ND mice. Thus, no benefit of ND was observed on the restCa in HU mice (Fig 4).

### Effects of HU and Nandrolone on the contractile parameters in mouse skeletal muscle

We measured the contractile parameters in Sol and EDL muscles in the 4 experimental groups (4–5 animals per group). As expected, the Sol muscle was the most involved in the HU-induced modifications of these parameters. There was a not significant trend of reduction of TTP (the time needed to reach the peak of the single twitch) and HRT (the time needed by the muscle to relax after the single twitch), which may be in accord with the slow-to-fast transition in HU mice. In the vehicle-treated (HU-V) and ND-treated (HU-ND) animals, this effect was even more pronounced, suggesting no beneficial effect of ND on these parameters (Table 1). We do not have an explanation for the significant HRT worsening in HU-V vs. HU; however HRT is the only affected parameter and it is unlikely that the vehicle may have significantly biased the effects of ND. The peak twitch tension (Ptw, mN) and the maximal isometric tetanic force (Po, mM) were significantly reduced in HU animals compared to controls. These parameters were still significantly lower than control in HU-V and HU-ND mice again suggesting no beneficial effects of ND. When normalized with respect to muscle cross-sectional area, this trend was maintained but the effect was no more significant. This suggests that the reduction of force was mainly due to the reduction of muscle mass and that the contractile apparatus was still functional in spite of atrophy (Table 1). Overall, treatment with ND had little effect on HU-induced contractile changes. Nevertheless, it should be considered that the Sol muscles of HU-ND mice, randomly assigned to contraction studies, showed a MW slightly greater with respect to HU mice, but still significantly reduced compared to CTRL mice. Thus, we can say that ND did not ameliorate muscle contractile function in absence of atrophy recovery, but we cannot formulate any hypothesis regarding contractile function recovery in mice with recovered muscle weight.

**Table 1:** Effect of Nandrolone on the contractile properties of HU Soleus muscle.

			Groups		
	Control	HU	HU-Vehicle-treated	HU-ND-treated	Statistical significance
**Parameters**					
Muscle weight (mg)	10.6 ± 1.2	6.7 ± 0.2*	7.4 ± 0.4*	7.1 ± 0.3*	F = 7.892 P<0.005
TTP (ms)	36.4 ± 1.0	34.1 ± 1.6	32.9 ± 2.4	30.8 ± 1.2	F = 2.2418 P>0.05
HRT (ms)	42.1 ± 2.3	35.6 ± 2.0	31.7 ± 2.1*	31.4 ± 2.1*	F = 5.3426 P = 0.0128
Ptw (mN)	22.3 ± 1.6	13.6 ± 1.5*	14.1 ± 1.3*	15.8 ± 1.9*	F = 5.5842 P = 0.011
sPtw (kN/m^2^)	17.4 ± 2.2	15.8 ± 1.3	13.3 ± 0.8	16.9 ± 2.4	F = 0.8845 P>0.05
Po (mN)	189.9 ± 12.4	92.0 ± 20.7*	112.5 ± 6.8*	117.9 ± 12.9*	F = 8.7370 P = 0.002
sPo (kN/m^2^)	145.4 ± 8.8	104.5 ± 18.1	107.4 ± 8.0	125.4 ± 14.9	F = 1.8536 P>0.05

The columns from left to right indicate the measure of the different contractile parameters (TTP, time to peak; HRT, half-maximal relaxation time; Ptw, peak twitch tension; sPtw, specific twitch tension; Po, maximal isometric tetanic force; sPo, specific tetanic force) measured from isolated Sol muscle from Control (4 animals) and HU mice (4 animals), HU mice treated with the vehicle HU-V (4 animals) and HU mice treated with ND (5 animals). See text for details. Statistical analysis was performed using ANOVA followed by post-hoc Bonferroni t-test (*at least P<0.05 versus control).

### Effects of HU and Nandrolone on gene expression evaluated by Real Time-PCR in mouse skeletal muscle

Levels of expression of mRNA encoding for genes involved in skeletal muscle atrophy and plasticity have been assayed by Real Time-PCR.

**Fig 5:**
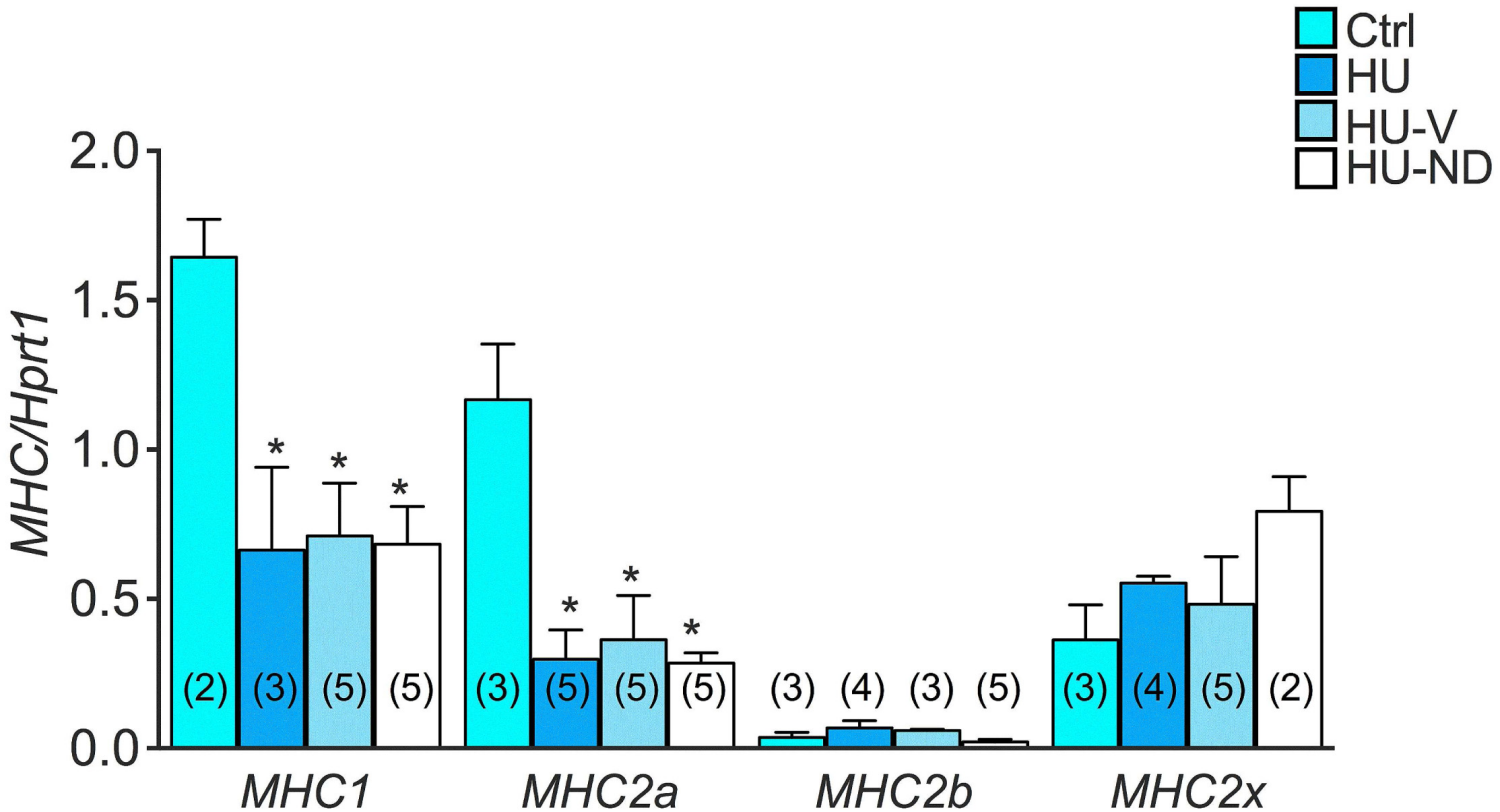
Effects of Nandrolone (ND) treatment on Myosin Heavy Chain (MHC) mRNA expression level in Soleus muscle of HU mice. Histograms show quantification of transcript levels performed with real time PCR, for different isotypes of *MHC* genes normalized by the *Hprt1* gene, in the 4 experimental groups (Ctrl, HU, HU-V, HU-ND). Each bar represents the mean ± S.E.M. from the number of animals as indicated in the brackets above the bars. Statistical analysis was performed for each muscle type using ANOVA followed by Fisher t-test *vs Ctrl (at least P<0.05).

We evaluated myosin heavy chain (MHC) isoform mRNA expression to estimate the effect of ND on the HU-induced slow-to-fast phenotype transition of Sol muscle. The mRNA amount of the 4 MHC isoforms (type 1, 2a, 2b, 2x) is modified during HU in accord with the variation of protein level, already measured in previous studies [12, 37, 39]. Indeed, we observed a significant reduction of the oxidative isoforms of MHC (type 1 and type 2a) mRNA and a slight and non-significant increase of the mRNA amount of the fast glycolytic ones (type 2b) (Fig 5). We found that ND did not restore the mRNA expression of the different types of MHC (Fig 5).

**Fig 6:**
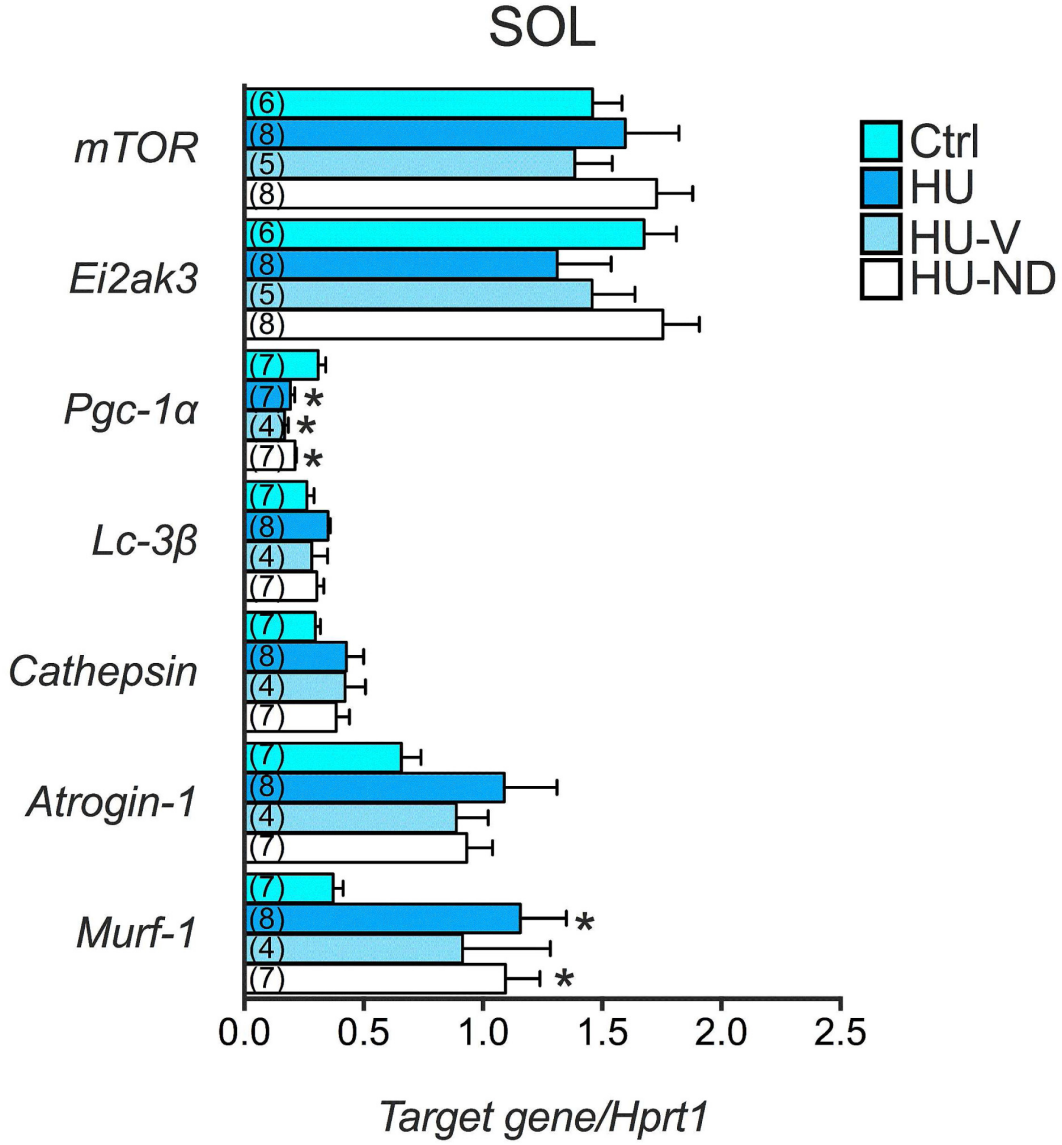
Effects of Nandrolone (ND) treatment on gene expression level in Soleus muscle of HU mice. Histograms show quantification of transcript levels performed with real time PCR, for *mTO*R, *Eif2ak3*, *Pgc1α*, *Lc3- β*, *Cathepsin*, *Atrogin-1 and Murf-1* genes normalized by the *Hprt1* gene, in the 4 experimental groups (Ctrl, HU, HU-V, HU-ND). Each bar represents the mean ± S.E.M. from the number of animals as indicated in the brackets above the bars. Statistical analysis was performed for each muscle type using ANOVA followed by Fisher t-test *vs Ctrl (at least P<0.05).

The slow-to fast phenotype transition was also evaluated in terms of PGC-1α expression level. This factor has been found to be highly expressed in oxidative (Sol) compared to glycolytic (EDL, Gas) muscles. The statistical analysis with the ANOVA test demonstrate a significant reduction of the expression of PGC1α in HU and HU-V groups compared to CTRL, accordingly with the phenotype transition. The treatment with ND had no effect, since PGC1α was significantly reduced also in HU-ND mice (Fig 6).

To explore the catabolic pathways, we evaluated two genes encoding for two muscle-specific ubiquitin ligases, MuRF-1 (Muscle-Ring Finger-1) and atrogin-1, which play a critical role in proteasome-dependent muscle proteolysis [41] and that are up-regulated in various conditions of muscle atrophy, including HU. In the present study, the expression of atrogenes was increased in Sol muscle of HU mice compared to CTRL and was not restored in HU-ND mice (Fig 6). In particular, MuRF-1 was significantly increased. We also evaluated the expression of two other genes coding for two markers of autophagy process, which are cathepsin-L, a lysosomal protease, and LC3-β, a protein involved in autophagosome formation. Activation of autophagy has been demonstrated in skeletal muscle during fasting and other conditions of muscle wasting. Little is known about the role of autophagy in the modifications found during HU. Here we found that LC3-β and cathepsin-L were not significantly modified in the 4 experimental groups (Fig 6).

We evaluated the mRNA expression amount of different genes involved in muscle anabolism, such as the eukaryotic translation initiation factor 2a kinase (PERK) and mTOR. We found that mTOR and PERK were not significantly modified in the 4 experimental conditions (Fig 6).

**Fig 7:**
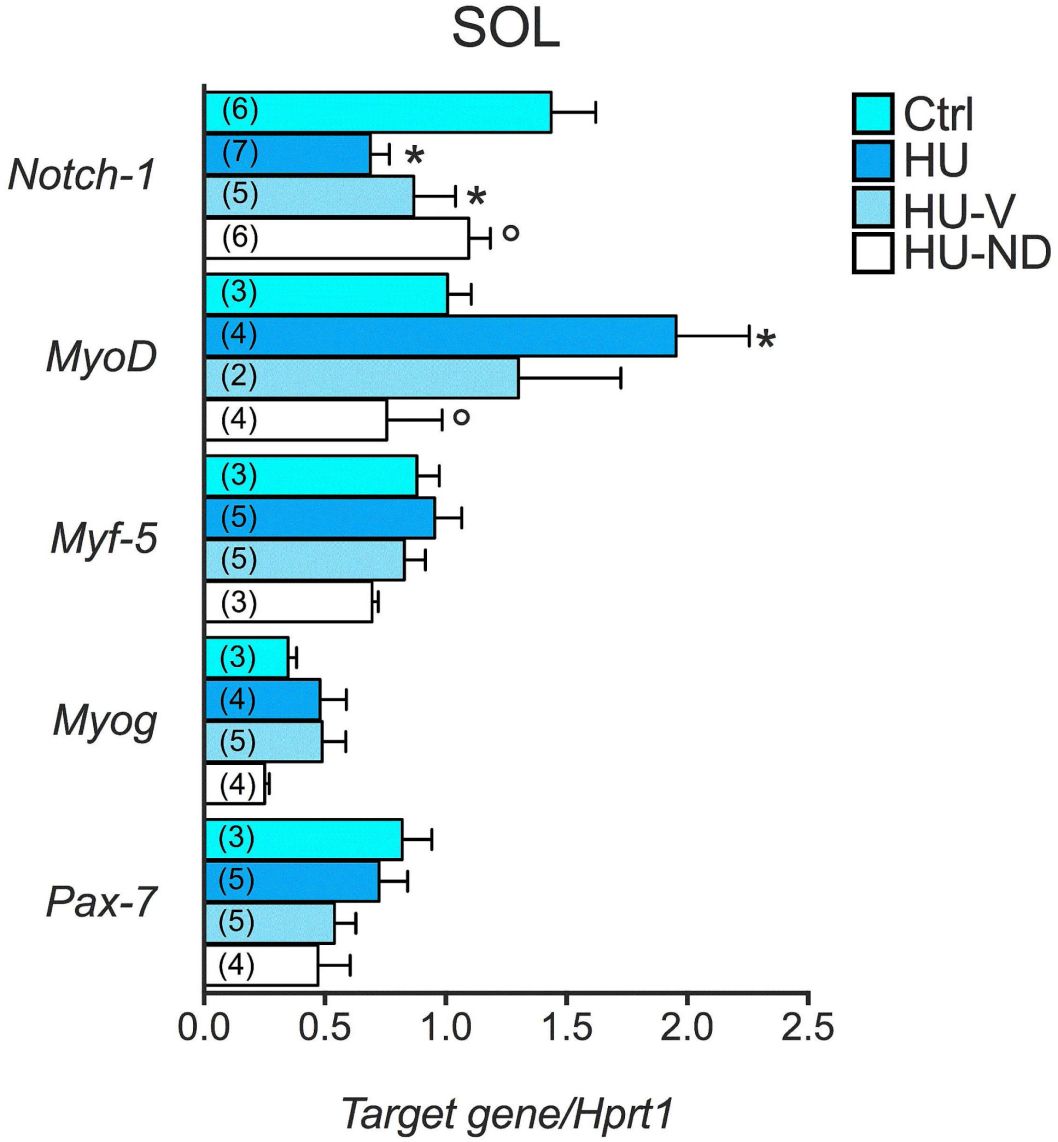
Effects of Nandrolone (ND) treatment on the expression level of genes involved in the regenerative pathways in Soleus muscle of HU mice. Histograms show quantification of transcript levels performed with real time PCR, for *Notch-1*, *MyoD*, *Myf5*, *Myogenin*, *Pax7* genes normalized by the *Hprt1* gene, in the 4 experimental groups (Ctrl, HU, HU-V, HU-ND). Each bar represents the mean ± S.E.M. from the number of animals as indicated in the brackets above the bars. Statistical analysis was performed for each muscle type using ANOVA followed by Fisher t-test *vs Ctrl, °vs HU (at least P<0.05).

We also estimated the regenerative potential in HU-ND muscles in terms of mRNA expression of specific markers of satellite cells activation, including Notch-1, Pax 7, Myf5, MyoD, myogenin [42, 43]. We found that MyoD mRNA was significantly increased after 14 days HU in Sol muscle, whereas Notch-1 was significantly down-regulated (Fig 7). Interestingly, ND treatment significantly re-establish MyoD and Notch-1 expression toward the control values. Myogenin, Pax-7 and Myf5 did not change significantly during HU and ND treatment (Fig 7).

No significant effect of HU or ND treatment was found on gene expression in EDL muscle (S3 Fig).

### Effects of HU and Nandrolone on eukaryotic initiation factor of protein translation (eIF2) evaluated by Western Blot in mouse skeletal muscle

**Fig 8:**
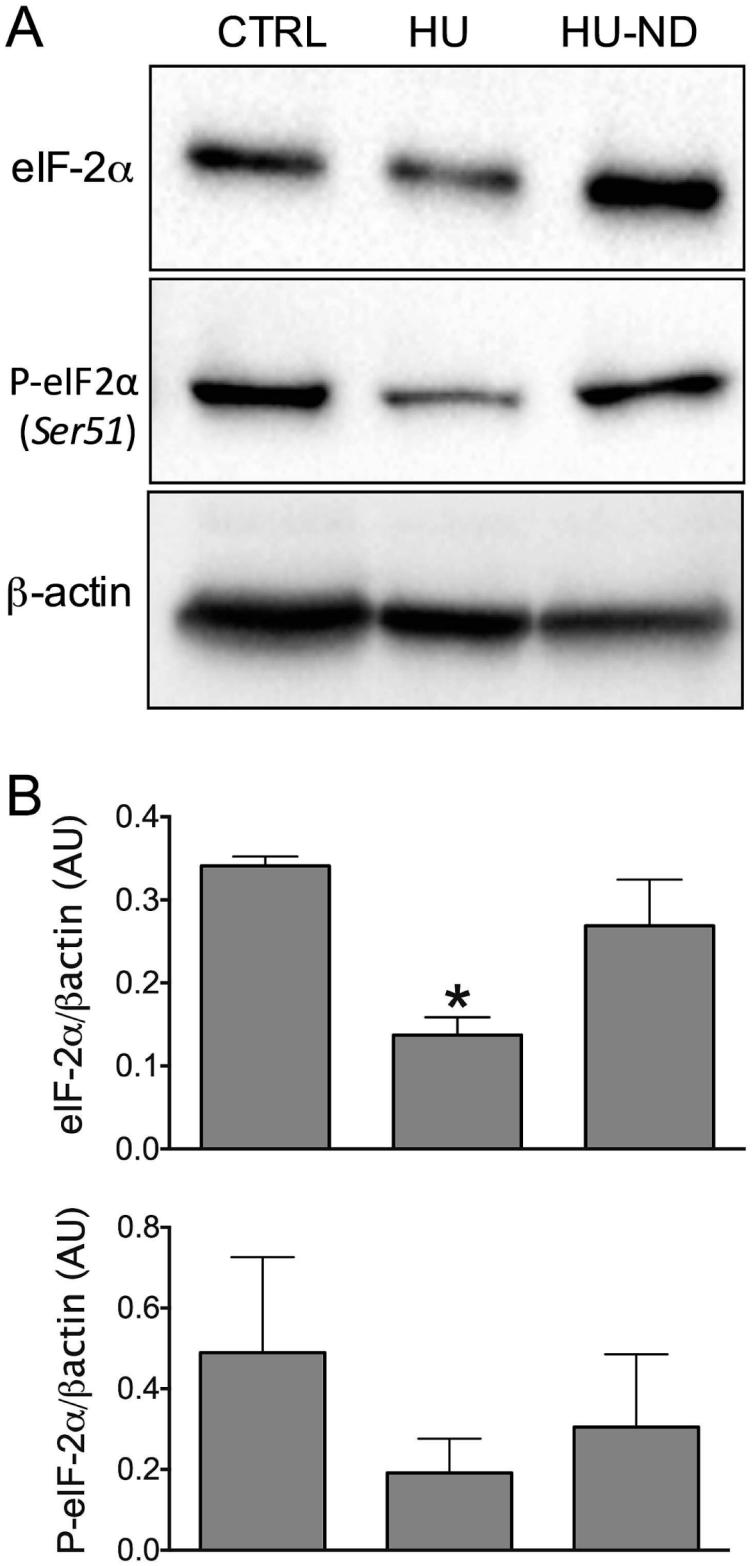
Effects of Nandrolone (ND) treatment on the expression level of protein involved in the anabolic pathways in Soleus muscle of HU mice. **A.** Western blot analysis of protein extracts prepared from muscle tissues. The blots were reacted with antibodies specific for eIF2α, eIF2α phosphorylated (Ser51) and β-actin, as indicated. The densitometric analysis of each experimental band was performed using Image Lab Analysis Software which detect the chemiluminescent signal intensity. **B.** Histograms show quantification of relative protein levels of bands found in Sol muscles of 2 CTRL, 3 HU and 3 ND-treated HU mice. Relative intensity was calculated by normalization of the absolute intensity of target protein with the absolute intensity of β-actin, as reference standard, and are represented as arbitrary units (AU). Each bar represents the mean ± S.E.M.. Statistical analysis was performed for each muscle type using ANOVA followed by Bonferroni (F = 6.34, P<0.05, 2/5) *Significantly different with respect to CTRL (P<0.05).

We measured the protein level of total eIF2 (eukaryotic Initiation Factor 2) and the phosphorylated form of eIF2, which are involved in the muscle anabolism. This factor, required for the initiation of translation, was found to be significantly reduced during HU and restored by ND treatment. Indeed, total eIF2 was reduced by 60 ± 6% during HU with respect to control value and increased in HU-ND by 96 ± 36% with respect to HU (Fig 8). These results suggest an involvement of ND also in the initiation of protein synthesis, through eIF2 increased expression. However, the phosphorylated form of eIF2 was not significantly modified in the various experimental conditions (Fig 8) suggesting that the eIF2 phosphorylation was less affected by ND treatment.

## Discussion

Under disuse conditions in rodents, there is a progressive loss of muscle mass which depends both on muscle type and on the duration of unloading and inactivity period [1, 14, 44]. Morphological changes with atrophy include a decreased cross-sectional area of muscle fibers, concomitant to a reduced whole muscle volume and mass, but no decrease in number of fibers [45]. The Sol muscle of rodents (both rats and mice), is a postural muscle highly susceptible to unloading [12, 46]. This muscle undergoes a shift in contractile properties of the fibers toward a faster phenotype, with a decrease in the time to peak tension and in the half-relaxation time of the twitch [47]. Although different drugs have been shown to ameliorate the phenotype transition and then muscle function, at the moment there are no efficient pharmaceutical options to prevent the loss of muscle mass or to enhance its recovery following an atrophy-inducing event. In an attempt to counteract the HU-related alterations of postural muscles, we have previously tested the effects of various compounds, such as trolox, insulin-like growth factor-1 (IGF-1) and taurine [12, 19, 31]. Although the slow-to-fast transition was counteracted to some extent, no beneficial effect was found on muscle atrophy.

Among the drugs potentially able to counteract muscle atrophy, the anabolic compounds may be particularly relevant because of their ability to improve muscle mass. Different studies have proposed the use of anabolic steroids, such as testosterone and its analogue nandrolone (ND) to contrast muscle atrophy in various disease and disuse conditions [48, 49–51]. The use of ND produced a significant increases in lean body and muscle mass as well as an improvement of physical activity in HIV-infected persons or in hemodialyzed patients [52, 53]. In rodent, ND has been shown to improve muscle wasting in the mdx mouse model of Duchenne muscular dystrophy [34]. Also in the HU rat, previous studies demonstrated that ND may be able to counterbalance Sol muscle atrophy induced by HU, although at doses higher than therapeutic ones [24]. The anabolic effect is likely mediated by the binding to the androgen receptor (AR) that allows the activation of specific DNA nucleotide sequences in the nucleus, which influence the transcriptional activity of specific pro-anabolic genes. As a result of the anabolic effect, this compound, by inducing muscle hypertrophy, can ameliorate muscle wasting by increasing the protein synthesis [54].

Based on these findings, we tested the effects of ND treatment as a pharmacological option in skeletal muscle atrophy of mice undergoing HU. The treatment schedule was designed as to reach an equilibrium state of endogenous steroid production in treated animals before HU and to verify if ND treatment can prevent muscle atrophy and functional alterations. So we have pretreated mice for 2 weeks before HU and we have continued for the entire duration of HU period (4 weeks administration in total). Interestingly, we found that the treatment with ND has a positive effect, being able to counteract the drop of Sol muscle weight induced by HU. The counteraction of HU-induced atrophy by ND was confirmed with the finer measurement of total protein content. We have explored the effects of ND on molecular pathways involved in the modulation of protein synthesis. Extensive literature supports the role of the mammalian target of rapamycin (mTOR) in the regulation of skeletal muscle hypertrophy [55, 56]. It has been hypothesized that under unloading condition the reduction of protein synthesis may be the result of mTOR inhibition [41, 57, 58]. Another candidate for the inhibition of protein synthesis during unloading might be the eukaryotic initiation factor of protein translation (eIF2α), since an increased expression of PKR-like endoplasmic reticulum-associated protein kinase (PERK) has been found in mice after 3-months spaceflight [44]. In this study, we observed no significant changes in mRNA expression level of mTOR and PERK in HU and HU-ND mice. However, the total eIF2 protein expression was reduced during HU and was significantly ameliorated in HU-ND mice, while expression of phosphorylated eIF2 was not modified. These results suggest a positive effect of ND in the eIF2-dependent protein synthesis.

We also looked at the effect of ND on the protein catabolism pathways involved in HU-induced muscle wasting. We found that ND had no beneficial effects on expression of the proteasome-related ubiquitin ligases, MuRF-1 and atrogin. In addition no effect of HU or ND was found on the autophagosome markers, cathepsin-L and LC3β that seem to be not involved.

An interesting finding of our investigation regards the involvement of the regenerative pathway in HU and after ND treatment. We found that MyoD mRNA was significantly increased after 14 days HU in Sol muscle, according to previous reports in HU rats and mice [59, 60]. MyoD is a myogenic transcription factor expressed in differentiating satellite cells [61]. At the same time, the expression of Notch-1, which promotes satellite cell self-renewal, was significantly down-regulated by HU, likely to favor myogenic differentiation [62, 63]. Thus it is possible that, during HU, satellite cells may differentiate in an attempt to limit muscle atrophy. Interestingly, the ND treatment prevent changes in MyoD and Notch-1 expression. This is in accord with the increased Notch expression and signaling in skeletal muscle by testosterone in older men and aged mice [64, 65]. Moreover, an histological analysis indicated an important role of ND in determining a decrease in the total area of muscle damage in the regenerating diaphragm of ND-treated mdx dystrophic mice. This result was attributed to a reduction in non-muscle area due to ND action [34, 66, 67]. Whether the effect of ND on Notch-1 and MyoD observed in HU mice is a prerequisite for or a consequence of the prevention of atrophy by the steroid remains to be elucidated.

Though ND had positive effect on muscle atrophy and restoration of protein synthesis, there was no evidence for an effect of the steroid on the HU-induced changes in phenotype or muscle function. Indeed, no effect of treatment was observed on the resting gCl and the restCa level, which showed in the HU-ND Sol muscle values typical of a fast phenotype. Also the expression of PGC1α, a molecular marker of the slow phenotype that was reduced in HU mice, was not restored in ND-treated HU mice. It is important to underline that lack of effects on restCa and gCl was observed in the entire set of Sol muscles independently of the recovery of muscle weight. In HU condition, the slow-to-fast phenotype transition is at least partially related to a change in motor-neuron electrical activity [68]. Accordingly, a low frequency electrical stimulation in HU Sol muscle was shown to partially improve Sol muscle mass and the peak tetanic force [69]. We have shown that a reduced protein kinase C activity, which is dependent on electrical input, during the first three days of disuse accounts for the early reduction of gCl, which in turn may be involved in the molecular phenotype transition [15, 18]. Also a reduced activity of stretch-activated calcium channels may lead to the early reduction of restCa, which in turn may contribute to the slow-to-fast transition of gene expression program [16]. In previous studies, we found that the amino acid taurine, the anti-oxidant trolox, and IGF-1 were able to partially counteract the phenotype transition in HU-Sol. It is worth noting that the modulation of restCa and/or gCl by these compounds is likely involved in their beneficial effect on muscle phenotype [12, 19, 31]. In the present study, ND did not show any effect on the contractile parameters. This result is in contrast to a previous study showing beneficial effects of ND treatment on contractile parameters in HU rats [24]. It should be however underlined that the experimental procedures were different, including species used, treatment regimen, as well as the method for contraction measurement.

In conclusion, a treatment with ND was able to maintain normal Sol muscle weight and protein content in HU mice. The molecular analysis suggests that such an effect may involve an increase of eIF2 protein expression and stimulation of Notch-dependent regeneration process. Nevertheless, despite the saving of protein content, Sol muscle function was not recovered with ND treatment. Together with previous results, this suggests that recovery of muscle weight and function do not necessarily go together. It is worth to note that the protective effect of ND on muscle after denervation or immobilization may vary with time of onset and duration of treatment [21, 70], thus further studies may usefully address different treatment schedules. In our study, the missing effect of ND on muscle function in HU Sol muscle may stem from the lack of direct effect on the gCl and restCa, with the inability to reverse the slow-to-fast phenotype transition. Thus, we hypothesize that co-administration of the anabolic ND with compounds able to ameliorate the functional properties can be beneficial in the treatment of muscle disuse. For instance taurine, a safe aminoacid, already demonstrated to ameliorate the HU-induced phenotype transition, can be useful in the modulation of functional parameters, such as restCa, gCl and contractile properties. Moreover it is possible that the preservation of muscle weight by ND treatment during disuse may be useful to accelerate recovery of both muscle mass and function upon the return to normal weight bearing and appropriate stimulation frequency by the motor unit firing pattern of slow-twitch Sol muscle.

## Supporting Information

S1 Fig Effects of Nandrolone (ND) treatment on EDL muscle weight of HU mice. Box and whisker chart created on the basis of single (circles) EDL muscle weight of mice belonging to the 4 experimental groups (15 CTRL: control mice, 8 HU: hindlimb unloaded mice, 11 HU-V: vehicle-treated hindlimb unloaded mice; 13 HU-ND: nandrolone-treated hindlimb unloaded mice). Statistical analysis was performed for each muscle type using ANOVA followed by Bonferroni’s t-test showed no significant differences (F = 0.7, dF = 3/43, N.S.).
(TIF)

S2 Fig Effects of Nandrolone (ND) treatment on the resting chloride conductance (gCl) measured in Extensor Digitorum Longus (EDL) muscle of HU mice. Resting gCl was measured from the total resting conductance (gm) minus the resting potassium conductance (gK). Each bar represents the mean ± S.E.M. measured in 20–44 fibers from EDL muscles of mice belonging to the different experimental conditions (3 CTRL: control mice, 3 HU: hindlimb unloaded mice, 3 HU-V: vehicle-treated hindlimb unloaded mice; 5 HU-ND: nandrolone-treated hindlimb unloaded mice). Statistical analysis was performed for each muscle type using ANOVA followed by Bonferroni’s t-test. (F = 0.93, dF = 3/101, N.S. for EDL muscle). No significant differences were found.
(TIF)

S3 Fig Effects of Nandrolone (ND) treatment on gene expression level in Extensor Digitorum Longus (EDL) muscle of HU mice. Histograms show quantification of transcript levels performed with real time PCR, for *mTO*R, *Eif2ak3*, *Notch1*, *Pgc1α*, *Lc3- β*, *Cathepsin*, *Atrogin1 and Murf-1* genes normalized by the *Hprt1* gene, in the 4 experimental groups (Ctrl, HU, HU-V, HU-ND). Each bar represents the mean ± S.E.M. measured from the number of muscle samples as indicated in the brackets above the bars. Statistical analysis was performed for each muscle type using ANOVA followed by Fisher t-test. No significant differences were found.
(TIF)

S4 Fig Effects of Nandrolone (ND) treatment on Soleus Muscle weight in Control-ground mice. Muscle weight was measured in 3 animals per group. No effects of Nandrolone treatment was found in this experimental condition.
(TIF)

S1 Table Analysis of gene expression data by one way-ANOVA and Multiple Comparisons- Fisher's in Soleus muscles of CTRL, HU, HU-V and HU-ND mice. (DOCX)

S2 Table Body weight in ND-treated mice before and after 14-days HU. (DOCX)
