# Supplementary figures and images for: Effects of Nandrolone in the Counteraction of Skeletal Muscle Atrophy in a Mouse Model of Muscle Disuse: Molecular Biology and Functional Evaluation
Source: PLoS One. 2015 Jun 11;10(6):e0129686. doi: 10.1371/journal.pone.0129686 (PMC4466268; doi:10.1371/journal.pone.0129686)

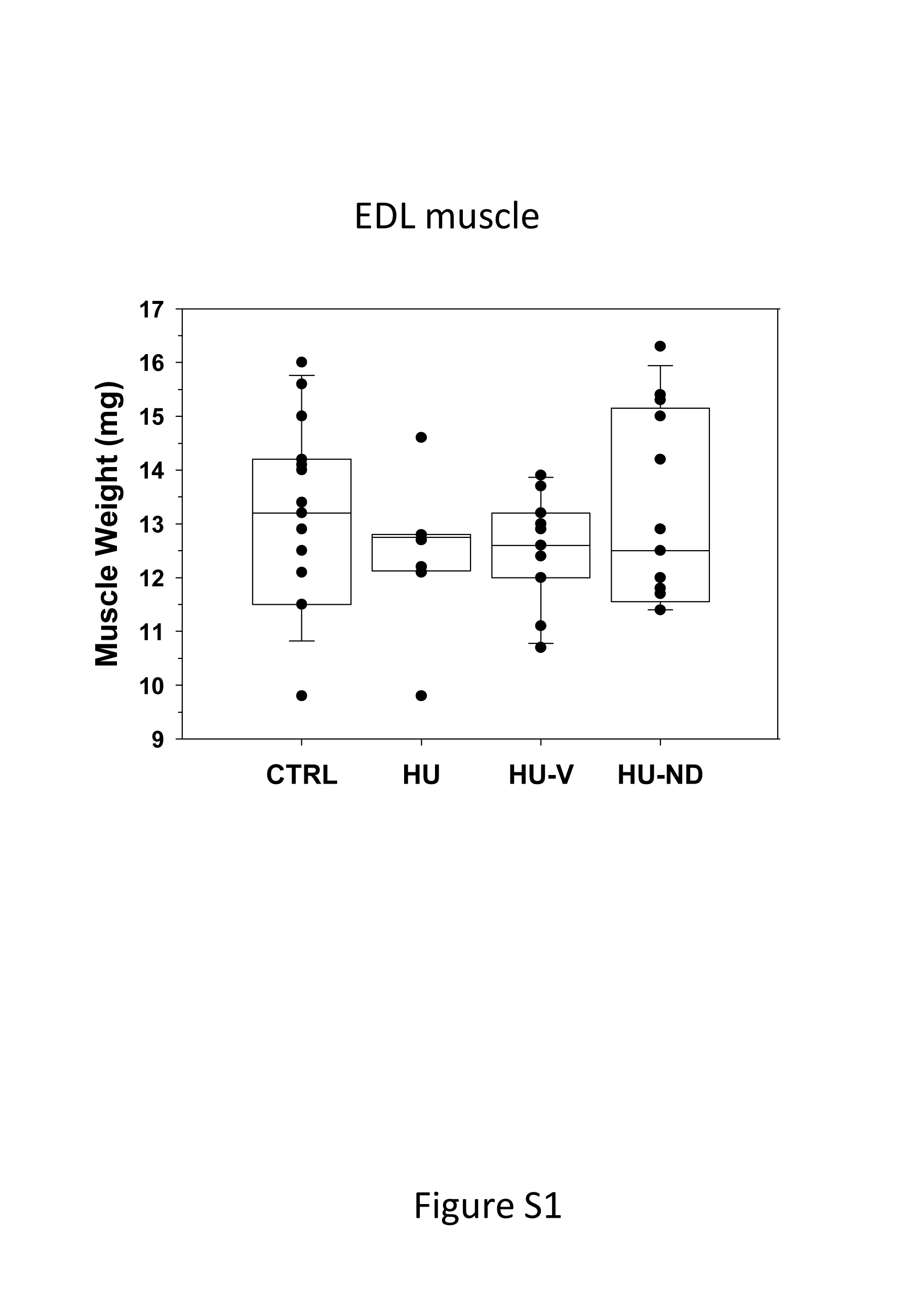

Supplement: S1 Fig — Box and whisker chart created on the basis of single (circles) EDL muscle weight of mice belonging to the 4 experimental groups (15 CTRL: control mice, 8 HU: hindlimb unloaded mice, 11 HU-V: vehicle-treated hindlimb unloaded mice; 13 HU-ND: nandrolone-treated hindlimb unloaded mice). Statistical analysis was performed for each muscle type using ANOVA followed by Bonferroni’s t-test showed no significant differences (F = 0.7, dF = 3/43, N.S.). (TIF) [file pone.0129686.s001.tif]

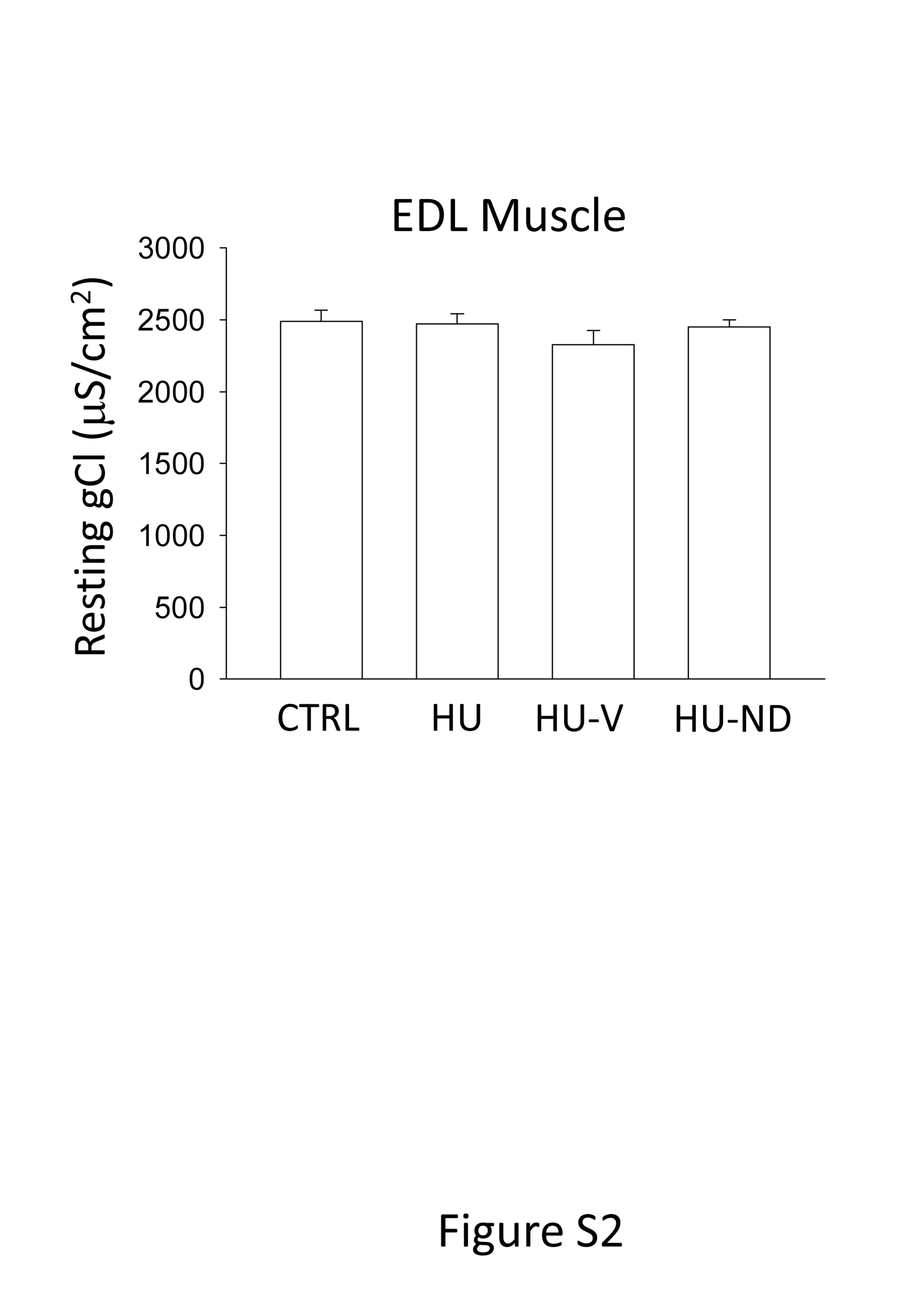

Supplement: S2 Fig — Resting gCl was measured from the total resting conductance (gm) minus the resting potassium conductance (gK). Each bar represents the mean ± S.E.M. measured in 20–44 fibers from EDL muscles of mice belonging to the different experimental conditions (3 CTRL: control mice, 3 HU: hindlimb unloaded mice, 3 HU-V: vehicle-treated hindlimb unloaded mice; 5 HU-ND: nandrolone-treated hindlimb unloaded mice). Statistical analysis was performed for each muscle type using ANOVA followed by Bonferroni’s t-test. (F = 0.93, dF = 3/101, N.S. for EDL muscle). No significant differences were found. (TIF) [file pone.0129686.s002.tif]

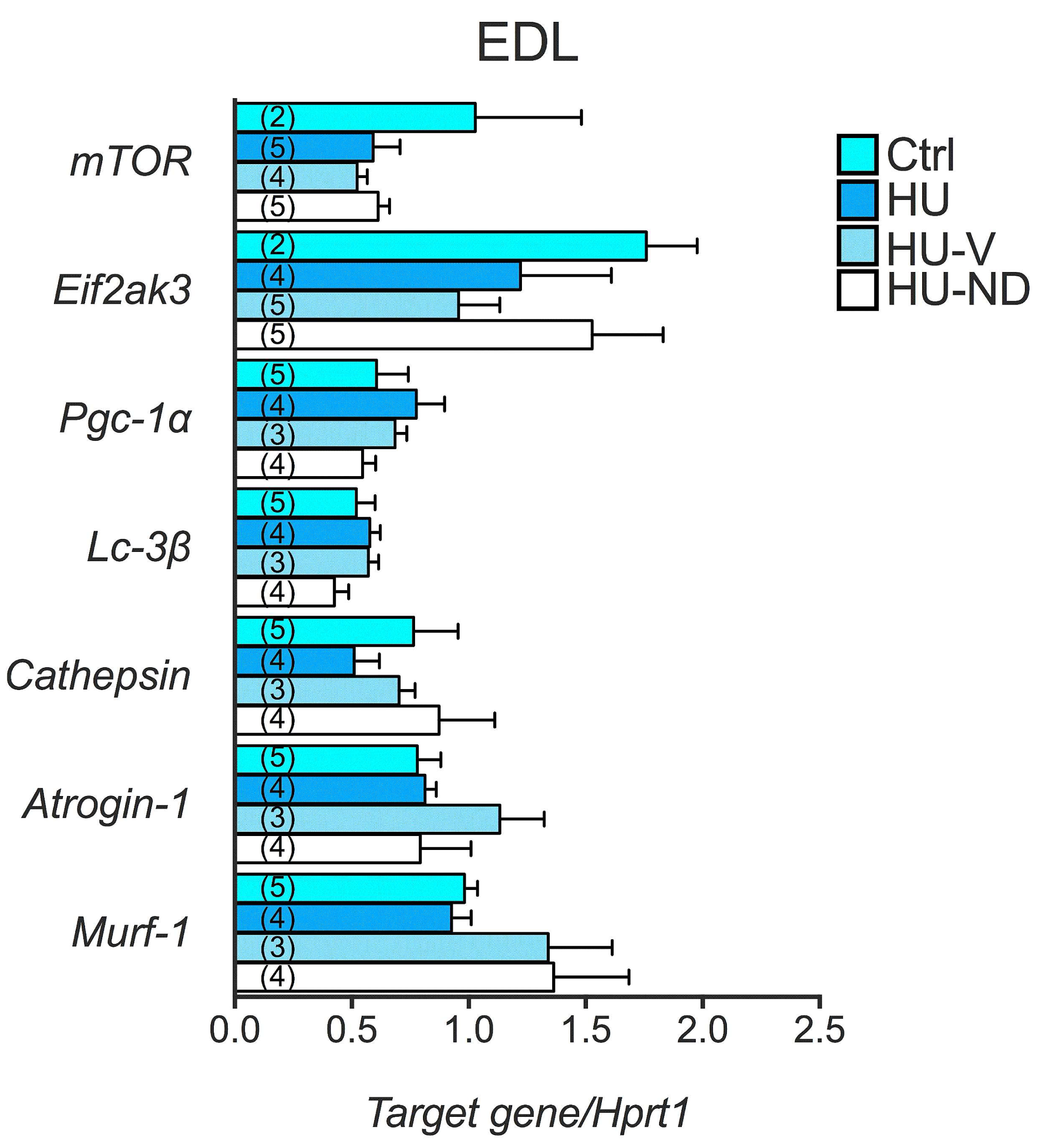

Supplement: S3 Fig — Histograms show quantification of transcript levels performed with real time PCR, for mTOR, Eif2ak3, Notch1, Pgc1α, Lc3- β, Cathepsin, Atrogin1 and Murf-1 genes normalized by the Hprt1 gene, in the 4 experimental groups (Ctrl, HU, HU-V, HU-ND). Each bar represents the mean ± S.E.M. measured from the number of muscle samples as indicated in the brackets above the bars. Statistical analysis was performed for each muscle type using ANOVA followed by Fisher t-test. No significant differences were found. (TIF) [file pone.0129686.s003.tif]

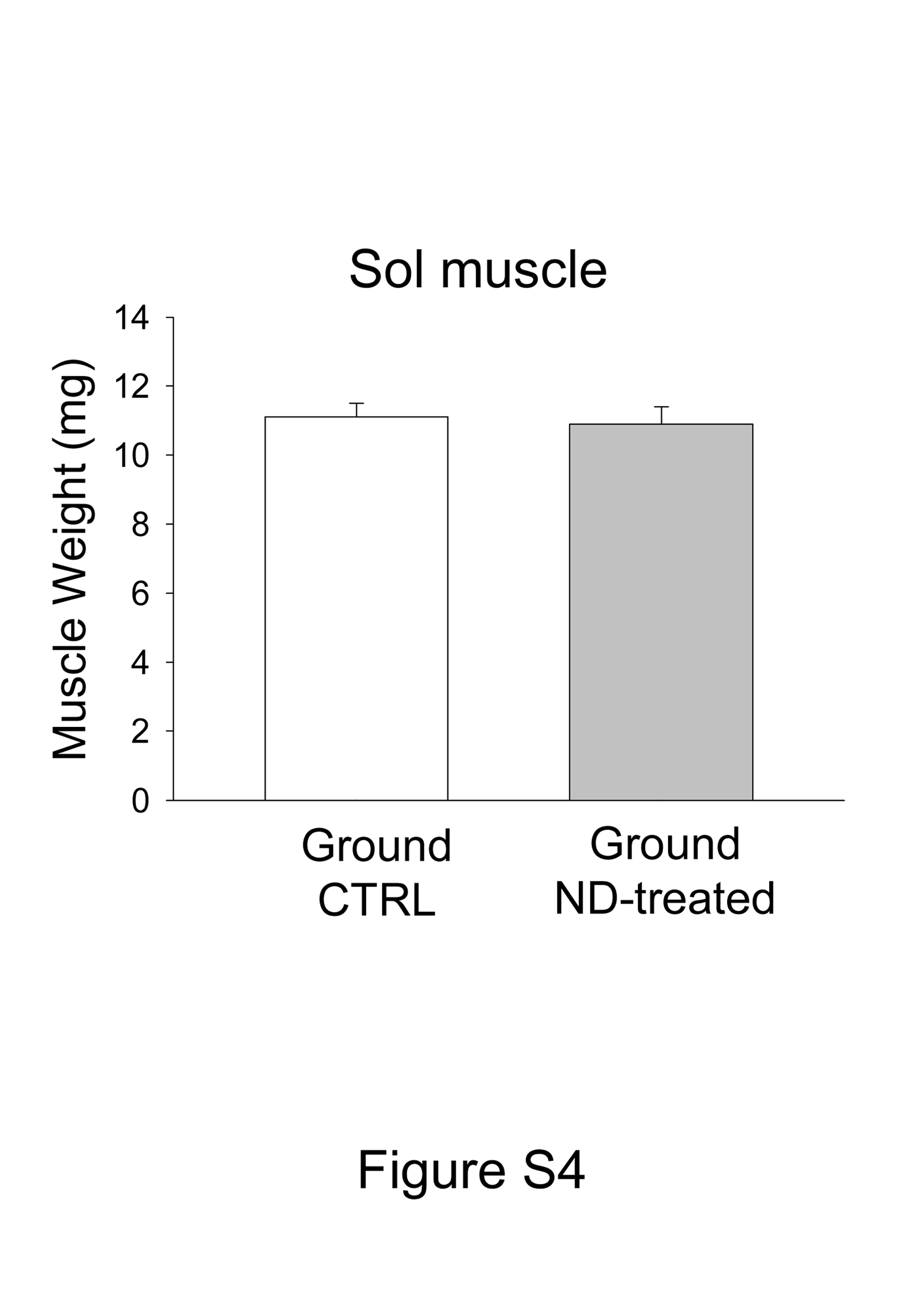

Supplement: S4 Fig — Muscle weight was measured in 3 animals per group. No effects of Nandrolone treatment was found in this experimental condition. (TIF) [file pone.0129686.s004.tif]
