# Supplementary material for: Effects of Nandrolone in the Counteraction of Skeletal Muscle Atrophy in a Mouse Model of Muscle Disuse: Molecular Biology and Functional Evaluation
Source: PLoS One. 2015 Jun 11;10(6):e0129686. doi: 10.1371/journal.pone.0129686 (PMC4466268; doi:10.1371/journal.pone.0129686)
Supplement: S1 Table — (DOCX) [file pone.0129686.s005.docx]

**Table S1. Analysis of gene expression data by one way-ANOVA and Multiple Comparisons- Fisher's in Soleus muscles of CTRL, HU, HU-V and HU-ND mice.**

| ***Gene*** | ***ANOVA*** | |  | ***P value Multiple Comparisons by Fisher's*** | | | | | |
| --- | --- | --- | --- | --- | --- | --- | --- | --- | --- |
|  | F | P | DF | HU vs CTRL | HU-V vs CTRL | HU-ND vs CTRL | HU-V vs HU | HU-ND vs HU | HU-ND vs HU-V |
| *Murf-1* | 4,308 | 0,0156 | 3/22 | 0,0033 | 0,0736 | 0,0077 | 0,4001 | 0,7981 | 0,5387 |
| *Atogin-1* | 1,391 | 0,2718 | 3/22 | - | - | - | - | - | - |
| *Cathepsin* | 1,021 | 0,4023 | 3/22 | - | - | - | - | - | - |
| *Lc-3* | 1,826 | 0,1719 | 3/22 | - | - | - | - | - | - |
| *Pgc-1* | 8,053 | 0,0009 | 3/21 | 0,0006 | 0,0005 | 0,003 | 0,4792 | 0,5114 | 0,211 |
| *Eif2ak3* | 1,326 | 0,2903 | 3/23 | - | - | - | - | - | - |
| *mTOR* | 0,673 | 0,5772 | 3/23 | - | - | - | - | - | - |
| *MHC1* | 4,046 | 0,0365 | 3/11 | 0,0127 | 0,0103 | 0,0088 | 0,8616 | 0,9419 | 0,9066 |
| *MHC 2a* | 9,904 | 0,0009 | 3/14 | 0,0003 | 0,0006 | 0,0002 | 0,6805 | 0,9361 | 0,6234 |
| *MHC 2b* | 2,11 | 0,1571 | 3/11 | - | - | - | - | - | - |
| *MHC 2x* | 1,259 | 0,3403 | 3/10 | - | - | - | - | - | - |
| *Notch-1* | 6,406 | 0,0032 | 3/20 | 0,0004 | 0,009 | 0,0829 | 0,3189 | 0,0305 | 0,2635 |
| *MyoD* | 4,384 | 0,0367 | 3/9 | 0,0318 | 0,5263 | 0,5173 | 0,1571 | 0,0071 | 0,2295 |
| *Myf-5* | 1,146 | 0,3701 | 3/12 | - | - | - | - | - | - |
| *Myog* | 1,853 | 0,1914 | 3/12 | - | - | - | - | - | - |
| *Pax-7* | 1,719 | 0,2122 | 3/13 | - | - | - | - | - | - |
