# Supplementary material for: Effects of Nandrolone in the Counteraction of Skeletal Muscle Atrophy in a Mouse Model of Muscle Disuse: Molecular Biology and Functional Evaluation
Source: PLoS One. 2015 Jun 11;10(6):e0129686. doi: 10.1371/journal.pone.0129686 (PMC4466268; doi:10.1371/journal.pone.0129686)
Supplement: S2 Table — (DOCX) [file pone.0129686.s006.docx]

**Table S2. Body weight in ND-treated mice before and after 14-days HU**

| Groups | Initial Body weight (g) | Final Body weight (g) |
| --- | --- | --- |
| CTRL | 29.2 ± 0.5 (15) | 30.3 ± 0.4 (15) |
| HU | 30.3 ± 0.4 (11) | 26.2 ± 1.0 (11)* |
| HU-ND-treated | 28.4 ± 0.5 (15) | 29.3 ± 0.4 (15) |

CTRL: control mice; HU: hindlimb unloaded mice; HU-ND treated: hindlimb unloaded mice treated with nandrolone. Significantly different by ANOVA F= 6.97 P<0.0001. followed by Bonferroni t-test *Significantly different vs. CTRLfinal body weight (P< 0.0001), vs. HU-ND-treated final body weight (P<0.005) and vs. HU initial body weight (P< 0.0001).
